# Hemostasis, bleeding and thrombosis in liver disease

**DOI:** 10.15761/JTS.1000182

**Published:** 2017-03-04

**Authors:** Brisas Flores, Hirsh D Trivedi, Simon C Robson, Alan Bonder

**Affiliations:** 1 Division of Internal Medicine, Beth Israel Deaconess Medical Center, Harvard University, 330 Brookline Avenue, 02215, Boston, USA; 2 Liver Center. Department of Medicine, Beth Israel Deaconess Medical Center, Harvard Medical School, Boston, MA, 02215, USA

**Keywords:** thrombosis, bleeding, hemorrhage, liver disease, cirrhosis, anticoagulation

## Abstract

The presence of cirrhosis poses an increased risk of both thrombosis and bleeding in individuals with chronic liver disease. This duality is a result of a dynamic disequilibrium between procoagulant and anticoagulant states in individuals with cirrhosis. The mechanism of this imbalance in cirrhosis remains unclear. It is known that the progression of cirrhosis leads to decreased synthetic function and a concurrent lack of natural anticoagulants. Other proposed mechanisms contributing to this hemostatic imbalance include decreased platelet production, increased platelet destruction from hypersplenism, decreased synthesis of Vitamin K-dependent and independent clotting factors and anticoagulant factors, and alterations in purinergic signaling pathways.

Given the current state of flux in our understanding of bleeding and thrombophilia in cirrhosis, the recommendations for treatment of these conditions are still evolving.

We provide a current update on the proposed pathophysiology of altered hemostasis and thrombophilia in cirrhosis. We discuss recent studies in portal vein thrombosis (PVT) and venous thromboembolism (VTE), which are the common thrombotic consequences of cirrhosis, resulting in substantive morbidity and mortality. To address these, we discuss new prophylactic interventions and current treatment options to manage the heightened risk of thrombosis in cirrhosis, while limiting hemorrhagic complications.

## Introduction

Patients with cirrhosis are at an increased risk for both bleeding and thrombosis. Multiple pathophysiologic changes occur in liver cirrhosis. The liver synthesizes coagulation factors, anticoagulants, proteins involved in fibrinolysis and the platelet production regulator, thrombopoeitin, from megakaryocytes [1,2]. Importantly, hepatic dysfunction perturbs the clotting process [1,2]. Portal hypertension leads to hypersplenism and shunting of blood into the peripheral circulation, which induces a consumptive coagulopathy further worsening thrombocytopenia [3,4]. Associated conditions, such as sepsis, nutritional deficiency, and other comorbid diseases, cause endothelial dysfunction and metabolic compromise, which can further disrupt hemostatic and clotting responses.

These mechanisms vary from patient to patient and risk should be assessed on an individualized basis. Unfortunately, standard coagulation tests are unreliable when stratifying bleeding or thrombotic risk in cirrhosis and may not be applicable in cirrhotic patients as they would in those with normal hepatic function [1,5–7]. Nonetheless, the risk of thrombosis should not be overlooked and anticoagulation should be employed when indicated for treatment.

## Coagulopathy *vs*. Thrombosis in Cirrhosis

**Figure 1:**
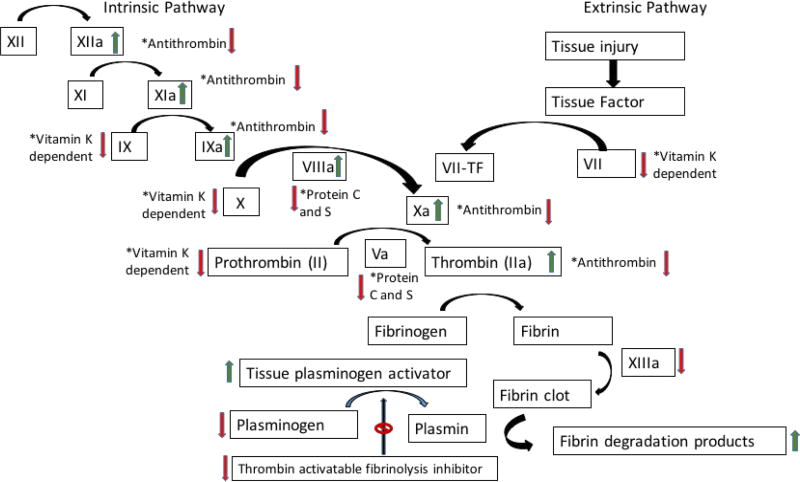
Review of coagulation cascade and the associated pathophysiologic changes that occur with cirrhosis [1,11–17]. The green arrow denotes products are increased, whereas the red arrow denotes products are decreased.

Cirrhosiscan lead to both a coagulopathic and procoagulant state [1]. There is decreased synthesis of Vitamin K-dependent and independent clotting factors and anticoagulants, platelet production abnormalities, and hypersplenism with platelet consumption [1–4]. Figure 1 describes the pathophysiologic changes that occur in cirrhosis.

The deficiency in vitamin K-dependent clotting factors (II, VII, IX and X) in those with cirrhosis results in an elevated international normalized ratio (INR). Although an elevated INR is interpreted as indicating one who is at an increased risk for bleeding, it is not the case for patients with cirrhosis and is not as reliable as in someone with normal liver function [8]. Similarly, a prolonged INR does not designate a protective role in development of hospital acquired deep venous thrombosis (DVT), and prothrombin time (PT) has not been predictive of bleeding from the gastrointestinal (GI) tract in cirrhosis [5]. Correlations between superficial bleeding and INR exist, but are inadequate [9]. In addition, there is little evidence to support that Vitamin K administration improves rates of bleeding or transfusion requirements in patients with cirrhosis [6,9].

Certain common medical interventions used to improve coagulopathy are controversial. Fresh frozen plasma (FFP) transfusions are often used in clinical practice before procedures in attempts to minimize bleeding risk. However, there is little evidence of improvement in coagulopathy with FFP. Larger volumes of transfusions only cause a transient improvement [7,10]. Desmopressin has been shown to increase levels of VIII and Von Willebrand factor (vWF), as well as improve bleeding time and PT in patients with cirrhosis, but there is no evidence that it decreases rates of bleeding or reduces transfusion requirements [9]. Factor VIIa has been found to transiently correct PT, but does not reliably prevent or control bleeding [11]. More randomized controlled trials are needed to better define the role of coagulation factor and recombinant activated factor VII administration for prophylaxis and treatment of hemorrhage in cirrhosis [11].

The regulation of clotting and thrombolysis is altered in those with cirrhosis. Natural anticoagulants, including antithrombin, inactivate thrombin and the active forms of Factors X, IX, XI, and XII [1]. Thrombomodulin makes thrombin less capable of cleaving fibrinogen when it is thrombin bound. Protein C and Protein S, which are also vitamin K-dependent zymogens, combine with thrombomodulin and other factors to inactivate Factor V and Factor VIII [1]. This interaction slows the effects of thrombin and reduces thrombosis [1,12].

As patients with cholestasis and other liver disease develop deficiency in vitamin K-dependent clotting factors, they also develop deficiency in Protein C and Protein S [13–17]. Patients with Child-Pugh Class C cirrhosis have been found to have as low as 40% Protein C concentration compared to those without cirrhosis [13]. In one study, Tripodi *et al.,* showed that factor VIII increases with higher Child-Pugh score and may be up to 200% greater in those with Child-Pugh C cirrhosis [13]. El Boki et al. found similar results, further validating these findings [14]. Tang *et al.,* found that Protein C, Protein S and antithrombin levels were progressively decreased with increasing severity of Child-Pugh class [15]. However, they did not find a difference in Factor VIII levels [15]. Despite the changes in Protein C, Protein S, and antithrombin, there was no difference in the rate of portal vein thrombosis development when adjusting for Child-Pugh score [15]. However, other evidence shows that Protein C and S are lower in cirrhotic patients with portal vein thrombosis when compared to those without [16].

Singhal *et al.,* also looked at Protein C and S, as well as antithrombin and factor V Leiden mutation in a total of 47 patients with end-stage cirrhosis: 89% had low levels of at least one, and 70% had low levels of all [17]. These deficiencies were greater in those with MELD >15 and also in those with hepatitis C infection [17].

## Fibrinolysis in Cirrhosis

Plasminogen and tissue plasminogen activator (t-PA) aid in the degradation of clots. Plasmin degrades Factor VIII, Factor V, vWF, and Factor XIII to impede coagulation, and also solubilizes fibrin to generate degradation products such as D-dimer, a marker for fibrin turnover and inflammation [1]. Tissue plasminogen activator is inhibited by plasminogen activator inhibitor type-1 (PAI-1), which is produced by endothelial cells [1,12]. Thrombin-activatable fibrinolysis inhibitor (TAFI), an enzyme made in the liver, prevents binding and activation of plasminogen thereby inhibiting fibrinolysis [1,12].

In patients with cirrhosis, high levels of D-dimer and t-PA were predictors of the first episode of gastrointestinal bleed [12]. Patients with cirrhosis have been found to have reduced plasma levels of plasminogen, alpha-2 antiplasmin, histidine-rich glycoprotein, and factor XIII, along with increased levels of t-PA [12]. Although TAFI has been found to be decreased in cirrhosis, with the hypothesis that this would lead to increased fibrinolysis, studies show conflicting results [12]. This may be secondary to reduction of profibrinolytic factors [12]. It has also been suggested that ascitic fluid contributes to systemic fibrinolysis through lymphatic drainage [18]. There is an association between high levels of endotoxin and markers of DIC, suggesting a relationship between clotting activation and endotoxemia [12].

## Thrombocytopenia

Thrombocytopenia occurs in cirrhosis as a result of increased splenic consumption and decreased platelet production. Thrombocytopenia has been related to increased bleeding risk, particularly with counts below 50,000 and in the setting of varices [3]. Thrombopoietin is the major regulator of platelet production [3]. Eltrombopag is a drug that was developed for promoting the production of platelets by operating at the thrombopoietin receptor, c-Mpl [3,19]. It was created with the intention to facilitate interferon and other antivirals for the treatment of Hepatitis C [19]. Afdhal, *et al.* (2012) studied the use of eltrombopag in patients with cirrhosis and thrombocytopenia who were to undergo an elective procedure and found that a large number of patients (104 out of 145) who received eltrombopag were able to avoid platelet transfusion, compared to only 28 out of 147 of those patients who received placebo (p<0.001) [19]. Although there was no difference in the rates of bleeding (17% of eltrombopag vs. 23% of placebo, p value not reported), there was a difference in rates of development of portal vein thrombosis (6 in eltrombopag vs. 1 in placebo, odds ratio 3.04, confidence interval 0.6–14.8) [19]. As a result of this adverse reaction, the study was terminated early [19].

Avatrombopag and romiplostim are two thrombopoietin receptor agonists, which are currently being investigated, that lead to enhancement of megakaryocytic proliferation and differentiation thus increasing platelet production [20]. Neither drug is yet approved for use [20]. Further research is necessary to validate these therapies.

## Purinergic signaling in cirrhosis

The extracellular nucleotides, adenosine triphosphate (ATP), adenosine diphosphate (ADP), uridine triphosphate (UTP), and uridine diphosphate (UDP) are important in liver mediated hemostasis. These nucleotides bind various type-2 purinergic/pyrimidinergic (P2Y1–14 and P2X1–7) receptors on platelets, endothelium, vascular smooth muscle and leukocytes [21]. ATP and ADP mediate hemostasis through the activation of platelet P2 receptors: P2Y12 and P2Y1. Moreover, ADP acts as a platelet recruiting factor. ATP, on the other hand, may be a competitive antagonist of ADP for platelet P2Y receptors [22–24]. Endothelial P2Y1 and P2Y2 activation by ATP and UTPleads to the release of prostacyclin (PGI2) and nitric oxide (NO), which can lead to both vasodilation and inhibition of platelet aggregation [25–27].

Ectonucleotidases are ecto-enzymes that regulate the P2-receptor-mediated effects; in particular, the CD39 family of ectonucleotidases. CD39 leads to the phosphohydrolysis of ADP and ATP, and therefore the generation of adenosine monophosphate (AMP) and adenosine. This leads to reduced platelet activation at the site of a clot. When injury occurs to the vasculature, vascular CD39 bioactivity is lost, allowing for local platelet activation [28]. CD39L1/NTPDase2, on the other hand, leads to platelet activation [28]. In the liver, CD39 is a thromboregulatory factor, whereas CD39L1 may be a hemostatic factor [29].

Purinergic signaling changes may lead to decreased or impaired endothelial dependent vascular relaxation, which may be partly responsible for the development or worsening of portal hypertension as cirrhosis progresses. The normal response to increased portal vein resistance is a decrease of hepatic artery resistance, but in cirrhosis this response is decreased [30]. Increased production of vasodilator molecules, such as nitric oxide, contributes to increased endothelium-dependent relaxation within the systemic and splanchnic arterial circulations resulting in a greater vasodilatory response of the hepatic artery to adenosine [30,31].

## Prophylaxis and treatment of venous thromboembolism in cirrhosis

Patients with cirrhosis are at increased risk for venous thromboembolism, specifically portal vein thrombosis. The prevalence of spontaneous portal vein thrombosis in patients with cirrhosis varies, and may be up to 26%, with some estimates reporting about 5–10% incidence each year [32,33]. The non-portal venous system thromboembolism incidence is anywhere from 0.5–8.1% in patients with cirrhosis, with increasing risk as Childs-Pugh score worsens [18,34]. Patients with portal vein thrombosis have an increased risk of variceal bleeding, ascites, intestinal ischemia, and mortality post-transplant [32,35].

There are two types of portal vein thrombosis: acute and chronic. Acute portal vein thrombosis is the sudden onset of portal occlusion due to thrombus. Patients may present with abdominal pain, variceal bleeding, intestinal infarction, or be asymptomatic [36]. Chronic portal vein thrombosis develops in patients who have acute portal vein thrombosis that does not resolve [36]. These patients will form collateral circulation to bring blood around the area of obstruction, a mechanism called cavernous transformation [36].

Despite their high risk of thrombosis, there is no consensus on prophylactic or therapeutic treatment with anticoagulation in patients with cirrhosis. Most people are treated if they have the diagnosis of an acute symptomatic PVT, the presence of another type of DVT, risk factors for thrombosis, or if they are pre-liver transplant patients [33]. In terms of treatment, options include no treatment, pharmacologic anticoagulation, and transjugular intrahepatic portal shunt (TIPS). Table 1 describes the mechanism of the anticoagulant medications described below and articles reviewed in this paper.

## Vitamin K antagonists

Vitamin K antagonists (VKAs), like warfarin, may be used for the treatment of PVT in patients with cirrhosis. Their use is encouraged over other anticoagulants because of convenience and physician comfort. Additionally, there is a lack of evidence for direct oral anticoagulants in this clinical context.

Warfarin is effective in preventing thrombus progression and improving recanalization rates [37]. However, there is no evidence to suggest that it improves hepatic decompensation rates or provides mortality benefit [37]. One study compared patients being treated for portal vein thrombosis with warfarin versus those who received no pharmacologic treatment. The mean INR was 1.38 in the treatment group versus 1.43 in the control group; there was no significant difference in the INRs between groups (p=0.571) [38]. When comparing those within the warfarin treatment group who achieved thrombus resolution to those who did not (termed “nonresponders”), nonresponders had a longer time from detection of PVT to treatment of PVT (p=0.03), thicker spleens (p=0.04), and lower platelet counts (p=0.06), but there was no difference in INR values (p=0.291) [38].

One caveat that arises in patients with cirrhosis is the elevated INR, which makes warfarin dosing challenging. There are limited studies evaluating goal INR for patients with cirrhosis on warfarin, making management difficult when choosing anticoagulation. A four-point scale was developed to identify patients with cirrhosis who would be high risk for warfarin use [39]. Patients receive one point if their albumin is 2.5–3.49 g/dL or if their creatinine is 1–1.99 mg/d and 2 points if albumin is less than 2.5 g/dL or if creatinine is greater than or equal to 2 mg/dL [39]. Those who have a score of zero have been found to have a lower time in the therapeutic range without an increase in bleeding [39]. However, those with cirrhosis and a score of four had poorer INR control and higher risk of bleeding [39]. For this reason, it is recommended to start at 1 mg and aim for a goal INR of 2–3[39]. The European Association for the Study of the Liver (EASL) recommends reduced warfarin dosing when baseline INR levels are elevated, with goal INR of 2–3[40].

## Low molecular weight heparin

Over the past few years, data has been emerging on the use of low molecular weight heparin (LMWH) in patients with cirrhosis. Treatment with LMWH can lead to complete recanalization in up to 45% of patients and lower rates of clot progression [33,34,41]. LMWH use for PVT prophylaxis and treatment is not associated with higher rates of bleeding, but treatment with unfractionated heparin may be [41–43].

Notably, it’s been found that prophylactic administration of LMWH to prevent portal vein thrombosis was effective without increasing the rate of bleeding [44]. Prophylactic use was also found to reduce rates of hepatic decompensation and bacterial translocation, suggesting a possible anti-inflammatory effect [44,45]. Other studies have suggested that prophylactic anticoagulation does not decrease rate of venous thromboembolism, but the authors did not comment on the development of PVT or if screening of PVT was done [42]. Some studies report increased minor bleeding risk with thromboprophylaxis in patients with cirrhosis, but with no increase in major bleeding or effect on mortality [46]. The patients included in these studies were older and had been hospitalized longer [46]. There are currently no recommendations for prophylactic widespread use of LMWH in cirrhosis.

## Direct oral anticoagulants

Although the recent decade has yielded evidence showing the utility and safety of direct oral anticoagulants (DOACs), evidence on use of DOACs in cirrhosis is lacking. Multiple studies have shown dabigatran, apixaban and rivaroxaban to be noninferior to warfarin in preventing stroke with no increased risk of major bleeding. However, Connoly et al. (2009) did show that when used for stroke prevention in atrial fibrillation, dabigatran dosed at 150 mg daily had higher rates of gastrointestinal bleeding compared to warfarin (p<0.001)[47]. Notably, many of the studies evaluating use of DOACs excluded patients with alanine aminotransferase and aspartate aminotransferase greater than two times the upper limit of normal, total bilirubin >1.5, hemoglobin <10 g/deciliter, platelets <100,000/cubic millimeteror gastrointestinal bleeding within six months to one year [47–50].

A recent study compared apixaban and rivaroxaban to traditional anticoagulation with warfarin and LMWH, and found there was no significant difference in rates of major bleeding or times until bleeding occurred (p=0.9)[51]. This study only included Child-Pugh A and B patients, and was small with only 39 cases reviewed [51]. There is concern that the hepatic and renal metabolism of rivaroxaban places patients with cirrhosis at higher risk of bleeding, but this has not been validated in clinical trials [52]. The cumulative evidence of the use of DOACs in liver disease is insufficient and concrete recommendations regarding their use in individuals with chronic liver disease cannot be made at this time.

## Transjugular intrahepatic portal shunt

Transjugular intrahepatic portal shunt (TIPS) has been adopted as a possible treatment option for PVT. The majority of studies evaluating TIPS in patient with cirrhosis have been for variceal bleeding and refractory ascites, rather than for the primary indication of PVT [53]. However, over the last decade or so, TIPS has demonstrated to be safe and effective in those with PVT [53].

In patients who do have PVT and varices, TIPS versus endoscopic band ligation has been studied for prevention of variceal bleeding [54]. In a small study of 25 patients, those who had TIPS had PVT improvement and lesser rates of rebleeding of varices (p=0.002)[54]. Survival rates were not different amongst the groups [54].

A study by Luca *et al.,* looked at patients treated with TIPS for complications of portal hypertension [55]. These patients were followed until last clinical evaluation, liver transplant, or death [55]. After TIPS, there was complete recanalization of PVT in 57% of patients, a decrease of thrombosis in 30%, and no improvement in 13%[55]. Of those who did achieve recanalization, 95% maintained a patent portal vein [55].

In a study by Senzolo *et al.,* TIPS was done in 28 patients, 13 of who were cirrhotic [56]. TIPS was performed for pre-transplantation, refractory ascites, variceal bleeding, portal biliopathy, and portal vein thrombosis complicating Budd–Chiari syndrome [56]. The study reports that the majority of those who had cirrhosis had improvement of Child-Pugh score after TIPS, with unreported statistical significance [56]. The main factor associated with success was related to the procedure itself and operator dependence, and a visible intrahepatic portal vein [56]. This highlights the importance of the operator and their experience with the procedure in these patients.

One question that arises is if patients with PVT who have TIPS also warrant anticoagulation after TIPS. Wang *et al.,* evaluated 64 patients treated with TIPS for PVT, where 31 patients received anticoagulation after TIPS and 33 did not [57]. The PVT were evaluated with cross-sectional CT scan for up to 12 months [57]. In 96.8% of these patients, thrombi improved after TIPS [57]. Recanalization rates were 83.9% versus 71.8% in patients treated with anticoagulation compared to those who were not [57]. This result was, however, not statistically significant (p=0.25)[57]. Clinical outcomes of gastrointestinal bleeding (p=0.67), shunt dysfunction (p=0.99), hepatic encephalopathy (p=0.71) and survival (p=0.99) were also similar between the groups [57]. Notably, the majority of these patients were treated with warfarin and the study had a relatively small sample size [57]. The use of LMWH in this patient cohort is still something to be investigated.

## Current guidelines and decision making

There is a paucity of guidelines regarding anticoagulation in cirrhosis for the treatment of PVT. The 2009 Practice Guidelines of the American Association for the Study of Liver Diseases (AASLD) do not make recommendations for or against anticoagulation in patients with cirrhosis and chronic PVT36. They do, however, recommend prior treatment of varices and GI bleeding prophylaxis before treatment with anticoagulation if these options were to be used [36].

In 2012, the American College of Chest Physicians recommended use of anticoagulation in cirrhotic patients with symptomatic splanchnic thrombosis, but not for incidental asymptomatic thrombosis [58]. The EASL 2016 Practice Guidelines recommend starting anticoagulation in patients with thrombosis for at least six months, with increased duration of treatment if PVT has extended, or if the patient is awaiting liver transplantation [40]. Similar to the 2009 AASLD guidelines, EASL recommends variceal bleeding prophylaxis prior to anticoagulation use [40]. EASL guidelines are in favor of using LMWH or warfarin as choice of anticoagulation [40]. Updated guidelines now recommend TIPS as well for pre-transplant patients [40]. However, no guidelines are present for prophylaxis in any patient with cirrhosis, including those who are pre-transplant. Recommendations for the use of aspirin are lacking.

## Recommendations

Our recommendations are as follows for patients with cirrhosis and thrombosis: all patients should have the diagnosis of PVT confirmed with cross-sectional imaging using computed tomography (CT) or magnetic resonance imaging (MRI). Patient should have an esophagogastroduodenoscopy (EGD) for the treatment of potential gastroesophageal varices prior to initiating anticoagulation in order to decrease the risk of first or recurrent bleed. Endoscopic band ligation can be used for treatment of esophageal varices, whereas endoscopic sclerotherapy or TIPS can be considered for gastric variceal decompression prior to initiating anticoagulation. We also make this recommendation for those with other thrombotic disorders other than PVT, including DVT, stable pulmonary embolism, or atrial fibrillation. However, the risks of delaying anticoagulation for EGD should be considered on an individual case-by-case basis.

**Figure 2:**
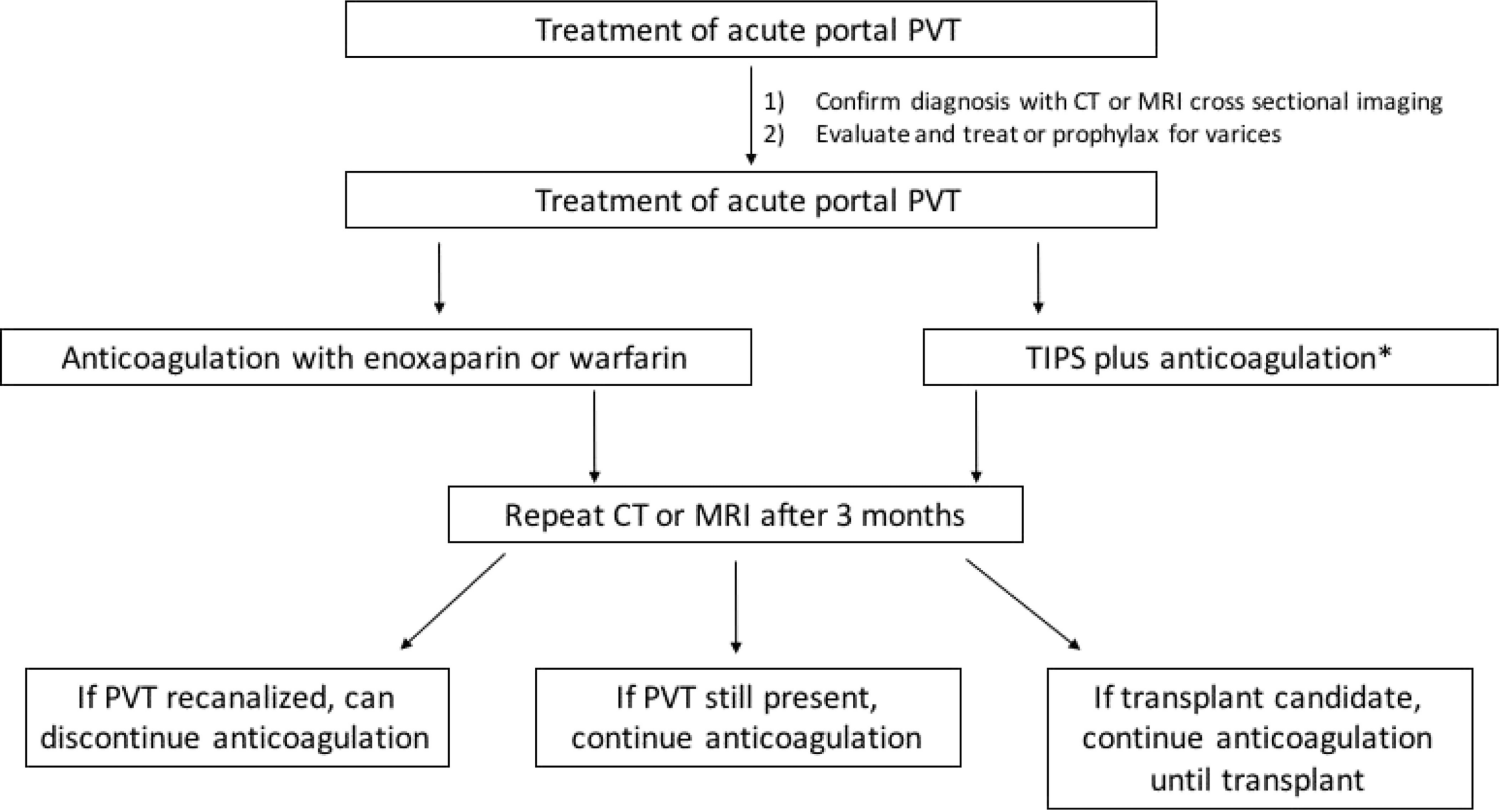
Recommended algorithm for treatment of acute portal vein thrombosis. *TIPS only to be employed if other indications for TIPS present.

As seen in the algorithm described in Figure 2, if the PVT is acute, then anticoagulation should be started using either LMWH or warfarin. Repeat imaging with CT or MRI, whichever was used for initial diagnosis, should be done at three months. Anticoagulation should be continued if recanalization is not achieved or if the patient is a transplant candidate. TIPS can be considered if there are other indications for TIPS present, such as refractory ascites, inability to control gastrointestinal variceal bleeding, etc. However, under these circumstances, anticoagulation should be continued after TIPS. Sending a procoagulant workup for further diagnostic and prognostic evaluation and management can be considered.

**Figure 3:**
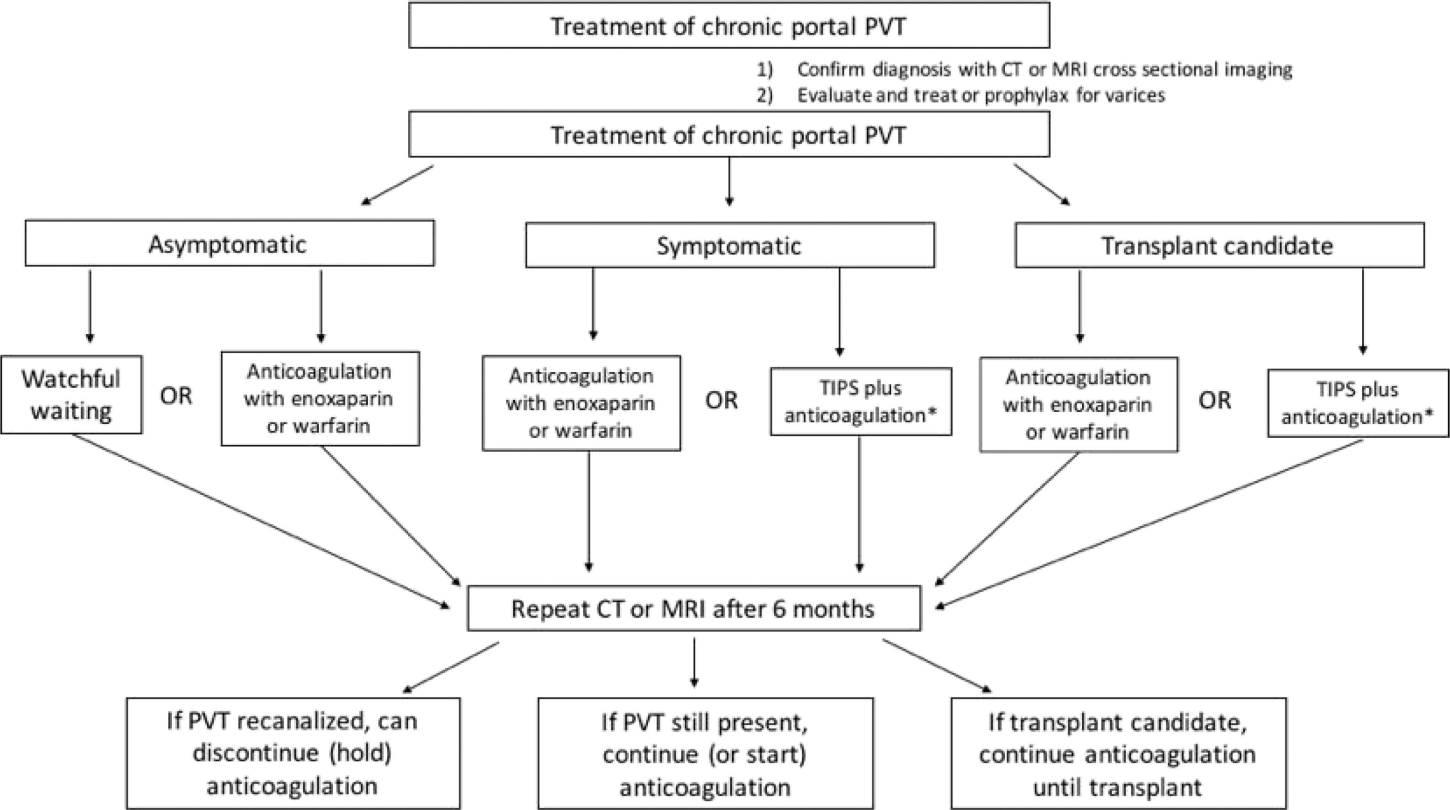
Recommended algorithm for treatment of chronic portal vein thrombosis. *TIPS only to be employed if other indications for TIPS present.

For the treatment of chronic PVT, shown by the algorithm in Figure 3, we recommend dividing treatment groups into symptomatic PVT (i.e. those with acute onset of pain, bleeding, ascites, or encephalopathy as result of PVT), asymptomatic PVT and transplant candidates. For those with symptomatic PVT, patients should be treated with LMWH or warfarin for at least six months. Cross-sectional imaging should be repeated after 6 months of anticoagulation to evaluate for recanalization. Anticoagulation should be extended for those who have not achieved recanalization or in those who are transplant candidates. Patients with asymptomatic PVT can either be treated similar to those with symptomatic PVT or can be monitored and treatment initiated if there’s extension of PVT. Transplant candidates should be treated with LMWH or warfarin until transplant. TIPS plus anticoagulation may also be utilized in this population if there are indications for TIPS present.

At this time, we are unable to make a recommendation for or against use of direct oral anticoagulants as data is still lacking. We do not recommend use of pharmacologic prophylaxis for PVT with these agents, but do recommend continuing standard DVT prophylaxis in hospitalized patients as indicated.

## Conclusions

Patients with liver cirrhosis are at increased risk for bleeding and thrombosis. It is difficult to predict which outcome cirrhotic patients may have. Multiple pathophysiologic changes occur in the setting of cirrhosis, and each patient’s risk should be assessed on an individualized basis with decisions made accordingly. Importantly, the risk of clotting and major complications should not be overlooked and anticoagulation should be employed when indicated.

## Figures and Tables

**Table 1:** Describes anticoagulants, respective mechanism of action, and reviewed articles in this paper [38,42–45,47].

Anticoagulant	Mechanism of action	Treatment or prophylaxis of PVT/Thromboembolism
Unfractionated heparin	Potentiates antithrombin III to inactivate thrombin; prevents conversion of fibrinogen to fibrin	Shatzel^42^
Low molecular weight heparin	Potentiates antithrombin III to inactivate thrombin; inhibits factor Xa	Villa^44^,Amitrano^45^,Shatzel^42^, Cerini^43^
Vitamin K antagonists	Inhibits vitamin K epoxide reductase complex 1	Chung^38^,Cerini^43^
Direct thrombin inhibitors	Inactivate circulating and clot-bound thrombin	None
Direct factor Xa inhibitors	Inactivate circulating and clot-bound factor Xa	Intagliata^47^
